# Crystal structure of tetra­kis­[μ_2_-2-(di­methyl­amino)­ethano­lato-κ^3^ *N*,*O*:*O*]di-μ_3_-hydroxido-di­thio­cyanato-κ^2^ *N*-dichromium(III)dilead(II) di­thio­cyanate aceto­nitrile monosolvate

**DOI:** 10.1107/S2056989016003996

**Published:** 2016-03-11

**Authors:** Julia A. Rusanova, Valentyna V. Semenaka, Irina V. Omelchenko

**Affiliations:** a Department of Chemistry, Taras Shevchenko National University of Kyiv, 64/13, Volodymyrska Street, Kyiv 01601, Ukraine; b SSI "Institute for Single Crystals", National Academy of Sciences of Ukraine, 60 Lenina ave., Kharkiv 61072, Ukraine

**Keywords:** crystal structure, *N*,*N*′-di­methyl­ethano­lamine, heterometal Pb^II^/Cr^III^ complex

## Abstract

The tetra­nuclear complex cation of the title compound, [Cr_2_Pb_2_(NCS)_2_(OH)_2_(C_4_H_10_NO)_4_](SCN)_2_·CH_3_CN, lies on an inversion centre. The main structural feature of the cation is a distorted seco-norcubane Pb_2_Cr_2_O_6_ cage with a central four-membered Cr_2_O_2_ ring. The Cr^III^ ion is coordinated in a distorted octa­hedron, which involves two N atoms of one bidentate ligand and one thio­cyanate anion, two μ_2_-O atoms of 2-(di­methyl­amino)­ethano­late ligands and two μ_3_-O atoms of hydroxide ions. The coordination geometry of the Pb^II^ ion is a distorted disphenoid, which involves one N atom, two μ_2_-O atoms and one μ_3_-O atom. In addition, weak Pb⋯S inter­actions involving the coordinating and non-coordinating thio­cyanate anions are observed. In the crystal, the complex cations are linked through the thio­cyanate anions *via* the Pb⋯S inter­actions and O—H⋯N hydrogen bonds into chains along the *c* axis. The chains are further linked together *via* S⋯S contacts. The contribution of the disordered solvent aceto­nitrile mol­ecule was removed with the SQUEEZE [Spek (2015 ▸). *Acta Cryst.* C**71**, 9–18] procedure in *PLATON*. The solvent is included in the reported mol­ecular formula, weight and density.

## Abstract 2

The crystal structure of the novel Pb/Cr heterometallic complex with 2-(di­methyl­amino)­ethanol prepared by direct synthesis is reported.

## Chemical context   

There is considerable inter­est in polynuclear heterometallic complexes as a result of their potential for inter­esting physico­chemical properties such as magnetic (Gheorghe *et al.*, 2010 ▸), catalytic (Trettenhahn *et al.*, 2006 ▸) and useful light- and/or redox-induced functions (Balzani *et al.*, 2009 ▸). The inter­est currently paid to the synthesis of polynuclear trans­ition metal complexes is stimulated, in particular, by attempts to design and construct multicomponent systems. Despite of a lot of work already done in this field, a limited number of synthetic strategies have been developed to date. Spontaneous self-assembly of Schiff base ligands or rigid building blocks appears to be an extremely powerful tool for the construction of novel polynuclear assemblies incorporating metal atoms by utilizing the various coordination modes of small and flexible ligands (Buvaylo *et al.*, 2005 ▸; Kirillov *et al.*, 2005 ▸). Metal powders have been successfully applied in direct synthesis of coordination compounds to yield a number of novel monometallic (Babich *et al.*, 1996 ▸) and heterometallic complexes (Buvaylo *et al.*, 2005 ▸) of various composition, nuclearities and dimensionalities. This work is a continuation of our investigations in the field of direct synthesis of heterometallic coord­ination compounds based on spontaneous self-assembly, in which one of the metals is introduced as a powder (zero-valent state) and oxidized during the synthesis (Nesterov *et al.*, 2011 ▸), in particular the application of Reinecke’s salt in direct synthesis of heterometallic complexes (Nikitina *et al.*, 2008 ▸).
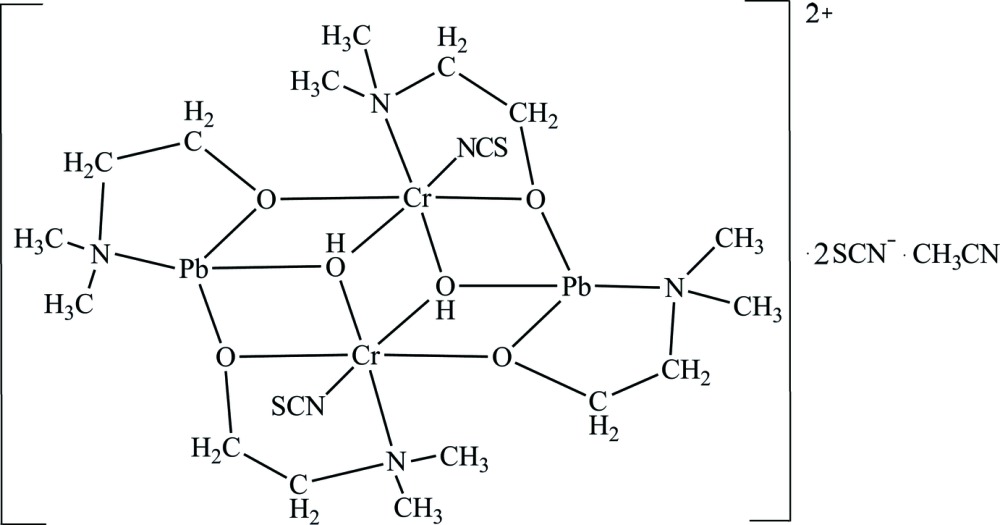


## Structural commentary   

**Figure 1:**
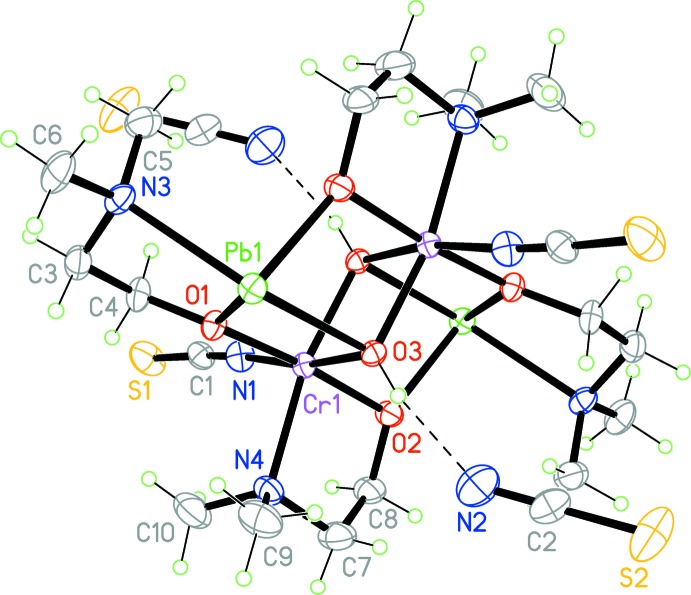
The mol­ecular structure of the title compound, shown with 30% probability displacement ellipsoids. O—H⋯N hydrogen bonds are shown as dashed lines.

The complex cation with a distorted seco-norcubane Pb_2_Cr_2_O_6_ framework is centrosymmetric, as shown in Fig. 1 ▸. The two crystallographically independent di­methyl­amino­ethanol ligands form five-membered chelate rings with the Cr^III^ and Pb^II^ ions. The Cr^III^ ion adopts a distorted octa­hedral coordination environment with one N atom and two μ_2_-O atoms from the di­methyl­amino­ethanol ligands and one μ_3_-O atom from the hydroxide ion in the equatorial plane, and one N atom of the thio­cyanate ion and one μ_3_-O atom of the second hydroxide ion in the axial positions. The Cr—O and Cr—N bond lengths are 1.950 (3)–1.993 (3) Å and 2.008 (4)–2.158 (4) Å, respectively, and the N—Cr—O and O—Cr—O angles are 79.10 (11)–93.48 (12)° for *cis*-positions and 168.63 (13)–173.46 (12)° for *trans*-positions. The Pb^II^ ion is tetra­coordinated by the one μ_3_-O atom of the hydroxide ion, one N atom and two μ_2_-O atoms of the di­methyl­amino­ethanol ligands and adopts a distorted disphenoidal coordination. There are additional weak Pb⋯S inter­actions [Pb1⋯S1 3.2749 (14) Å and Pb1⋯S2 3.4056 (16) Å], and thus the coordination geometry of the Pb^II^ ion can be considered as a strongly distorted trigonal prism, if these inter­actions are included. The Pb—O bond lengths [2.308 (3)–2.686 (3) Å] as well as the Pb—N distance [2.547 (4) Å] are in a good agreement with literature values. In general, all geometric parameters of the title complex cation are in good agreement with those in related amino­alcohol complexes (Shahid *et al.*, 2011 ▸).

## Supra­molecular features   

**Figure 2:**
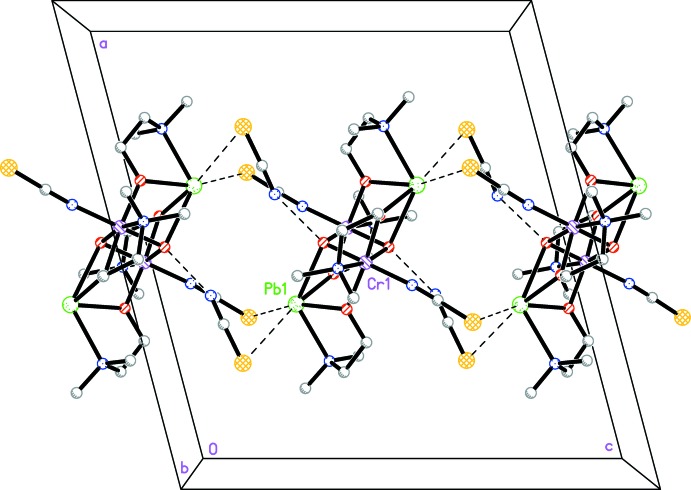
Crystal packing diagram of the title compound, viewed along the *b* axis. Pb⋯S contacts and O—H⋯N hydrogen bonds are shown as dashed lines.

In the crystal, the tetra­nuclear complex cations are linked through thio­cyanate anions with the above-mentioned inter­molecular Pb⋯S inter­actions and by an O—H⋯N hydrogen bond (Table 1 ▸) into chains along the *c* axis (Fig. 2 ▸). The chains are further linked together by an S⋯S sigma-hole bond [S1⋯S2 3.585 (2) Å], where atom S2 acts as a lone-pair donor.

## Database survey   

A search of the Cambridge Structural Database (Version 5.36; last update February 2015; Groom & Allen, 2014 ▸) for related complexes with 2-di­methyl­amino­ethanol gave 260 hits, including some closely related structures with a distorted seco-norcubane cage with Ti (Hollingsworth *et al.*, 2008 ▸), Ge(Sn)–Li (Khrustalev *et al.*, 2004 ▸, 2008 ▸ *)* and Na(Li)–Al (Nöth *et al.*, 2001 ▸).

## Synthesis and crystallization   

Lead monoxide (0,279 g, 1.25 mmol), NH_4_[Cr(NCS)_4_(NH_3_)_2_]·H_2_O (0.443 g, 1.25 mmol), NH_4_SCN (0.095 g, 1.25 mmol), 2-dimethylaminoethanol (0.5 ml, 5 mmol) and aceto­nitrile (20 ml) were heated in air at 323–333 K and stirred for 110 min until complete PbO dissolution occurred. Dark-grey crystals suitable for the crystallographic study were formed by slow evaporation of the resulting solution in air. The crystals were filtered off, washed with dry isopropyl alcohol and finally dried *in vacuo* at room temperature. Yield: 0.11 g, 10.3%.

The IR spectrum of the title compound (as KBr pellets) exhibited absorbance at 2250 cm^−1^ assigned to υ(CN) of the solvent aceto­nitrile mol­ecule, as well two additional bands at 2080 cm^−1^ and 1610 cm^−1^, which were assigned, respectively, to stretch and vibrational υ(CN) modes of the SCN anion. Analysis calculated for C_22_H_45_Cr_2_N_9_S_4_Pb_2_: C 22.43, H 3.85, N 10.69, S 10.88%; found: C 22.21, H 3.78, N 10.45, S 10.64%.

## Refinement   

Crystal data, data collection and structure refinement details are summarized in Table 2 ▸. All hydrogen atoms were placed in idealized positions and refined as riding, with *U*_iso_(H) = 1.2*U*_eq_(C) or 1.5*U*_eq_(C,O) for methyl and hydroxyl groups.

During the refinement, several isolated electron density peaks were located, which were assignable to a solvent acetnitrile mol­ecule(s) from the IR data and elementary analysis. Satisfactory results (*R*_1_ = 0.045) were obtained modeling the disordered C and N atoms, but very large displacement parameters for them were observed. The SQUEEZE (Spek, 2015 ▸) procedure in *PLATON* (Spek, 2009 ▸) indicated solvent cavities of volume 118 Å^3^ centered at (0.5, 0, 0.25), (0.5, 0, 0.75), (0, 0.5, 0.75) and (0, 0.5, 0.25), each containing approximately 18 electrons. In the final refinement, this contribution was removed from the intensity data, producing better refinement results. We assumed full occupancy of the solvent mol­ecule for each cavity, although the estimated 18 electrons are fewer than the 22 electrons expected for full occupancy. The solvent mol­ecule is included in the reported mol­ecular formula, weight and density.

## Supplementary Material

Crystal structure: contains datablock(s) I, global. DOI: 10.1107/S2056989016003996/is5439sup1.cif

Structure factors: contains datablock(s) I. DOI: 10.1107/S2056989016003996/is5439Isup2.hkl

CCDC reference: 1460850

Additional supporting information:  crystallographic information; 3D view; checkCIF report

## Figures and Tables

**Table 1:** Hydrogen-bond geometry (Å, °)

*D*—H⋯*A*	*D*—H	H⋯*A*	*D*⋯*A*	*D*—H⋯*A*
O3—H3⋯N2	0.82	1.95	2.757 (6)	169

**Table 2:** Experimental details

Crystal data	
Chemical formula	[Cr_2_Pb_2_(NCS)_2_(OH)_2_(C_4_H_10_NO)_4_](NCS)_2_·C_2_H_3_N
*M*_r_	1178.29
Crystal system, space group	Monoclinic, *C*2/*c*
Temperature (K)	298
*a*, *b*, *c* (Å)	17.533 (1), 13.8815 (7), 16.6179 (8)
β (°)	104.771 (6)
*V* (Å^3^)	3910.9 (4)
*Z*	4
Radiation type	Mo *K*α
μ (mm^−1^)	9.39
Crystal size (mm)	0.4 × 0.1 × 0.1

Data collection	
Diffractometer	Agilent Xcalibur Sapphire 3
Absorption correction	Multi-scan (*CrysAlis PRO*; Agilent, 2011 ▸)
*T*_min_, *T*_max_	0.382, 1.000
No. of measured, independent and observed [*I* > 2σ(*I*)] reflections	20193, 5680, 4133
*R*_int_	0.064
(sin θ/λ)_max_ (Å^−1^)	0.703

Refinement	
*R*[*F* ^2^ > 2σ(*F* ^2^)], *wR*(*F* ^2^), *S*	0.035, 0.058, 0.93
No. of reflections	5680
No. of parameters	190
H-atom treatment	H-atom parameters constrained
Δρ_max_, Δρ_min_ (e Å^−3^)	1.00, −0.69

Computer programs: *CrysAlis PRO* (Agilent, 2011 ▸), *SHELXS97* and *SHELXL97* (Sheldrick, 2008 ▸), *OLEX2* (Dolomanov *et al.*, 2009 ▸) and *PLATON* (Spek, 2009 ▸).

## supplementary crystallographic information

### Crystal data

**Table:**

[Cr_2_Pb_2_(NCS)_2_(OH)_2_(C_4_H_10_NO)_4_](NCS)_2_·C_2_H_3_N	*F*(000) = 2256
*M**_r_* = 1178.29	*D*_x_ = 2.001 Mg m^−^^3^
Monoclinic, *C*2/*c*	Mo *K*α radiation, λ = 0.71073 Å
*a* = 17.533 (1) Å	Cell parameters from 4335 reflections
*b* = 13.8815 (7) Å	θ = 2.9–32.5°
*c* = 16.6179 (8) Å	µ = 9.39 mm^−^^1^
β = 104.771 (6)°	*T* = 298 K
*V* = 3910.9 (4) Å^3^	Block, metallic dark gray
*Z* = 4	0.4 × 0.1 × 0.1 mm

### Data collection

**Table:**

Agilent Xcalibur Sapphire 3 diffractometer	5680 independent reflections
Radiation source: Enhance (Mo) X-ray Source	4133 reflections with *I* > 2σ(*I*)
Graphite monochromator	*R*_int_ = 0.064
Detector resolution: 16.1827 pixels mm^-1^	θ_max_ = 30.0°, θ_min_ = 3.0°
ω scans	*h* = −24→24
Absorption correction: multi-scan (*CrysAlis PRO*; Agilent, 2011)	*k* = −19→18
*T*_min_ = 0.382, *T*_max_ = 1.000	*l* = −23→23
20193 measured reflections	

### Refinement

**Table:**

Refinement on *F*^2^	Primary atom site location: structure-invariant direct methods
Least-squares matrix: full	Secondary atom site location: difference Fourier map
*R*[*F*^2^ > 2σ(*F*^2^)] = 0.035	Hydrogen site location: inferred from neighbouring sites
*wR*(*F*^2^) = 0.058	H-atom parameters constrained
*S* = 0.93	*w* = 1/[σ^2^(*F*_o_^2^) + (0.0118*P*)^2^] where *P* = (*F*_o_^2^ + 2*F*_c_^2^)/3
5680 reflections	(Δ/σ)_max_ = 0.001
190 parameters	Δρ_max_ = 1.00 e Å^−^^3^
0 restraints	Δρ_min_ = −0.69 e Å^−^^3^

### Special details

**Table:**

Geometry. All e.s.d.'s (except the e.s.d. in the dihedral angle between two l.s. planes) are estimated using the full covariance matrix. The cell e.s.d.'s are taken into account individually in the estimation of e.s.d.'s in distances, angles and torsion angles; correlations between e.s.d.'s in cell parameters are only used when they are defined by crystal symmetry. An approximate (isotropic) treatment of cell e.s.d.'s is used for estimating e.s.d.'s involving l.s. planes.
Refinement. Refinement of *F*^2^ against ALL reflections. The weighted *R*-factor *wR* and goodness of fit *S* are based on *F*^2^, conventional *R*-factors *R* are based on *F*, with *F* set to zero for negative *F*^2^. The threshold expression of *F*^2^ > σ(*F*^2^) is used only for calculating *R*-factors(gt) *etc*. and is not relevant to the choice of reflections for refinement. *R*-factors based on *F*^2^ are statistically about twice as large as those based on *F*, and *R*- factors based on ALL data will be even larger.

### Fractional atomic coordinates and isotropic or equivalent isotropic displacement parameters (Å^2^)

**Table:**

	*x*	*y*	*z*	*U*_iso_*/*U*_eq_	
Pb1	0.369714 (9)	0.456817 (12)	0.330204 (9)	0.03296 (5)	
Cr1	0.46177 (4)	0.59683 (5)	0.51003 (4)	0.02815 (15)	
S1	0.33608 (10)	0.73222 (11)	0.70835 (8)	0.0651 (4)	
S2	0.75622 (10)	0.58870 (14)	0.31923 (11)	0.0865 (6)	
O1	0.35991 (16)	0.5544 (2)	0.44114 (16)	0.0327 (7)	
O2	0.56441 (17)	0.6487 (2)	0.56837 (17)	0.0365 (7)	
O3	0.50477 (15)	0.52564 (19)	0.42849 (15)	0.0294 (6)	
H3	0.5314	0.5554	0.4027	0.044*	
N1	0.4136 (2)	0.6465 (3)	0.5993 (2)	0.0412 (9)	
N2	0.6095 (3)	0.6076 (4)	0.3499 (3)	0.0735 (14)	
N3	0.2425 (2)	0.4171 (3)	0.3704 (2)	0.0385 (9)	
N4	0.4503 (2)	0.7343 (3)	0.4474 (2)	0.0410 (9)	
C1	0.3816 (3)	0.6803 (3)	0.6461 (3)	0.0395 (11)	
C2	0.6716 (3)	0.6005 (4)	0.3384 (3)	0.0513 (13)	
C3	0.2214 (3)	0.5127 (3)	0.3976 (3)	0.0441 (12)	
H3A	0.1746	0.5067	0.4181	0.053*	
H3B	0.2094	0.5561	0.3504	0.053*	
C4	0.2877 (2)	0.5549 (3)	0.4655 (3)	0.0402 (11)	
H4A	0.2746	0.6205	0.4770	0.048*	
H4B	0.2939	0.5176	0.5161	0.048*	
C5	0.2516 (3)	0.3465 (4)	0.4382 (3)	0.0536 (13)	
H5A	0.2014	0.3357	0.4499	0.080*	
H5B	0.2882	0.3708	0.4871	0.080*	
H5C	0.2710	0.2870	0.4217	0.080*	
C6	0.1806 (3)	0.3839 (4)	0.2987 (3)	0.0568 (14)	
H6A	0.1336	0.3700	0.3160	0.085*	
H6B	0.1980	0.3267	0.2762	0.085*	
H6C	0.1697	0.4332	0.2568	0.085*	
C7	0.5302 (3)	0.7792 (4)	0.4780 (3)	0.0604 (15)	
H7A	0.5637	0.7591	0.4429	0.072*	
H7B	0.5253	0.8488	0.4747	0.072*	
C8	0.5662 (3)	0.7508 (3)	0.5642 (3)	0.0510 (13)	
H8A	0.6202	0.7736	0.5813	0.061*	
H8B	0.5371	0.7786	0.6010	0.061*	
C9	0.4340 (4)	0.7257 (4)	0.3548 (3)	0.0702 (17)	
H9A	0.4295	0.7889	0.3305	0.105*	
H9B	0.3855	0.6913	0.3338	0.105*	
H9C	0.4764	0.6916	0.3407	0.105*	
C10	0.3883 (3)	0.7963 (4)	0.4640 (4)	0.0733 (18)	
H10A	0.3869	0.8559	0.4344	0.110*	
H10B	0.3992	0.8090	0.5226	0.110*	
H10C	0.3382	0.7645	0.4459	0.110*	

### Atomic displacement parameters (Å^2^)

**Table:**

	*U*^11^	*U*^22^	*U*^33^	*U*^12^	*U*^13^	*U*^23^
Pb1	0.03289 (9)	0.03895 (10)	0.02546 (8)	−0.00140 (8)	0.00458 (6)	−0.00003 (8)
Cr1	0.0277 (4)	0.0296 (4)	0.0261 (3)	0.0001 (3)	0.0050 (3)	−0.0017 (3)
S1	0.0878 (12)	0.0615 (10)	0.0580 (8)	0.0261 (8)	0.0407 (8)	0.0061 (7)
S2	0.0671 (11)	0.1125 (15)	0.0917 (12)	−0.0349 (10)	0.0421 (10)	−0.0433 (11)
O1	0.0271 (15)	0.0382 (17)	0.0324 (14)	−0.0022 (12)	0.0070 (12)	−0.0052 (13)
O2	0.0353 (17)	0.0310 (17)	0.0376 (15)	−0.0028 (13)	−0.0009 (13)	0.0001 (13)
O3	0.0298 (15)	0.0320 (17)	0.0285 (13)	−0.0004 (12)	0.0111 (12)	0.0022 (12)
N1	0.044 (2)	0.044 (2)	0.0354 (19)	0.0010 (18)	0.0085 (18)	−0.0085 (18)
N2	0.075 (4)	0.085 (4)	0.069 (3)	−0.007 (3)	0.033 (3)	0.004 (3)
N3	0.032 (2)	0.044 (2)	0.0366 (19)	−0.0103 (17)	0.0048 (17)	−0.0009 (18)
N4	0.042 (2)	0.036 (2)	0.040 (2)	0.0025 (17)	0.0008 (18)	0.0030 (18)
C1	0.044 (3)	0.035 (3)	0.037 (2)	0.004 (2)	0.007 (2)	−0.004 (2)
C2	0.060 (4)	0.059 (4)	0.037 (2)	−0.017 (3)	0.015 (3)	−0.004 (2)
C3	0.031 (2)	0.052 (3)	0.047 (3)	0.002 (2)	0.007 (2)	0.002 (2)
C4	0.026 (2)	0.052 (3)	0.044 (2)	0.002 (2)	0.011 (2)	−0.008 (2)
C5	0.056 (3)	0.049 (3)	0.057 (3)	−0.007 (2)	0.018 (3)	0.007 (3)
C6	0.035 (3)	0.077 (4)	0.052 (3)	−0.017 (3)	−0.001 (2)	−0.006 (3)
C7	0.061 (4)	0.046 (3)	0.066 (3)	−0.015 (3)	0.001 (3)	0.011 (3)
C8	0.045 (3)	0.034 (3)	0.065 (3)	−0.011 (2)	−0.002 (3)	0.001 (2)
C9	0.086 (5)	0.068 (4)	0.048 (3)	0.009 (3)	0.003 (3)	0.019 (3)
C10	0.081 (4)	0.051 (4)	0.089 (4)	0.025 (3)	0.025 (4)	0.015 (3)

### Geometric parameters (Å, º)

**Table:**

Pb1—O2^i^	2.308 (3)	C3—H3A	0.9700
Pb1—O1	2.329 (3)	C3—H3B	0.9700
Pb1—N3	2.547 (4)	C4—H4A	0.9700
Pb1—O3	2.686 (3)	C4—H4B	0.9700
Cr1—O1	1.950 (3)	C5—H5A	0.9600
Cr1—O2	1.951 (3)	C5—H5B	0.9600
Cr1—O3	1.975 (3)	C5—H5C	0.9600
Cr1—O3^i^	1.993 (3)	C6—H6A	0.9600
Cr1—N1	2.008 (4)	C6—H6B	0.9600
Cr1—N4	2.158 (4)	C6—H6C	0.9600
S1—C1	1.627 (5)	C7—C8	1.465 (6)
S2—C2	1.603 (6)	C7—H7A	0.9700
O1—C4	1.424 (5)	C7—H7B	0.9700
O3—Cr1^i^	1.993 (3)	C8—O2	1.419 (5)
O3—H3	0.8211	C8—H8A	0.9700
N1—C1	1.167 (5)	C8—H8B	0.9700
N2—C2	1.157 (6)	C9—H9A	0.9600
N3—C6	1.467 (5)	C9—H9B	0.9600
N3—C5	1.471 (5)	C9—H9C	0.9600
N3—C3	1.479 (6)	C10—H10A	0.9600
N4—C10	1.466 (6)	C10—H10B	0.9600
N4—C9	1.497 (6)	C10—H10C	0.9600
N4—C7	1.498 (6)	O2—Pb1^i^	2.308 (3)
C3—C4	1.515 (6)		
			
O2^i^—Pb1—O1	85.15 (10)	N3—C3—H3B	109.3
O2^i^—Pb1—N3	88.84 (11)	C4—C3—H3B	109.3
O1—Pb1—N3	70.85 (10)	H3A—C3—H3B	107.9
O2^i^—Pb1—O3	65.32 (9)	O1—C4—C3	110.8 (3)
O1—Pb1—O3	62.83 (8)	O1—C4—H4A	109.5
N3—Pb1—O3	127.79 (9)	C3—C4—H4A	109.5
O1—Cr1—O2	173.46 (12)	O1—C4—H4B	109.5
O1—Cr1—O3	84.20 (11)	C3—C4—H4B	109.5
O2—Cr1—O3	93.48 (12)	H4A—C4—H4B	108.1
O1—Cr1—O3^i^	98.58 (11)	N3—C5—H5A	109.5
O2—Cr1—O3^i^	86.94 (11)	N3—C5—H5B	109.5
O3—Cr1—O3^i^	79.10 (11)	H5A—C5—H5B	109.5
O1—Cr1—N1	92.45 (13)	N3—C5—H5C	109.5
O2—Cr1—N1	90.82 (14)	H5A—C5—H5C	109.5
O3—Cr1—N1	170.07 (13)	H5B—C5—H5C	109.5
O3^i^—Cr1—N1	92.21 (13)	N3—C6—H6A	109.5
O1—Cr1—N4	91.44 (13)	N3—C6—H6B	109.5
O2—Cr1—N4	82.75 (13)	H6A—C6—H6B	109.5
O3—Cr1—N4	96.70 (13)	N3—C6—H6C	109.5
O3^i^—Cr1—N4	168.63 (13)	H6A—C6—H6C	109.5
N1—Cr1—N4	92.71 (15)	H6B—C6—H6C	109.5
C4—O1—Cr1	125.4 (2)	C8—C7—N4	110.6 (4)
C4—O1—Pb1	118.6 (2)	C8—C7—H7A	109.5
Cr1—O1—Pb1	113.50 (12)	N4—C7—H7A	109.5
Cr1—O3—Cr1^i^	100.90 (11)	C8—C7—H7B	109.5
Cr1—O3—Pb1	99.42 (10)	N4—C7—H7B	109.5
Cr1^i^—O3—Pb1	96.14 (10)	H7A—C7—H7B	108.1
Cr1—O3—H3	118.3	O2—C8—C7	107.9 (4)
Cr1^i^—O3—H3	124.4	O2—C8—H8A	110.1
Pb1—O3—H3	113.0	C7—C8—H8A	110.1
C1—N1—Cr1	174.3 (4)	O2—C8—H8B	110.1
C6—N3—C5	109.0 (4)	C7—C8—H8B	110.1
C6—N3—C3	109.9 (4)	H8A—C8—H8B	108.4
C5—N3—C3	110.6 (3)	N4—C9—H9A	109.5
C6—N3—Pb1	111.8 (3)	N4—C9—H9B	109.5
C5—N3—Pb1	114.4 (3)	H9A—C9—H9B	109.5
C3—N3—Pb1	100.9 (2)	N4—C9—H9C	109.5
C10—N4—C9	106.5 (4)	H9A—C9—H9C	109.5
C10—N4—C7	111.4 (4)	H9B—C9—H9C	109.5
C9—N4—C7	107.4 (4)	N4—C10—H10A	109.5
C10—N4—Cr1	114.3 (3)	N4—C10—H10B	109.5
C9—N4—Cr1	113.3 (3)	H10A—C10—H10B	109.5
C7—N4—Cr1	103.9 (3)	N4—C10—H10C	109.5
N1—C1—S1	177.1 (4)	H10A—C10—H10C	109.5
N2—C2—S2	177.9 (5)	H10B—C10—H10C	109.5
N3—C3—C4	111.8 (4)	C8—O2—Cr1	111.9 (3)
N3—C3—H3A	109.3	C8—O2—Pb1^i^	131.0 (3)
C4—C3—H3A	109.3	Cr1—O2—Pb1^i^	110.78 (13)
			
O2—Cr1—O1—C4	−126.9 (10)	O1—Pb1—N3—C3	33.2 (2)
O3—Cr1—O1—C4	163.7 (3)	O3—Pb1—N3—C3	61.4 (3)
O3^i^—Cr1—O1—C4	85.6 (3)	O1—Cr1—N4—C10	68.5 (4)
N1—Cr1—O1—C4	−7.0 (3)	O2—Cr1—N4—C10	−114.5 (4)
N4—Cr1—O1—C4	−99.7 (3)	O3—Cr1—N4—C10	152.8 (3)
O2—Cr1—O1—Pb1	71.6 (11)	O3^i^—Cr1—N4—C10	−139.6 (6)
O3—Cr1—O1—Pb1	2.16 (13)	N1—Cr1—N4—C10	−24.0 (4)
O3^i^—Cr1—O1—Pb1	−75.86 (13)	O1—Cr1—N4—C9	−53.7 (3)
N1—Cr1—O1—Pb1	−168.47 (15)	O2—Cr1—N4—C9	123.3 (4)
N4—Cr1—O1—Pb1	98.76 (15)	O3—Cr1—N4—C9	30.6 (3)
O2^i^—Pb1—O1—C4	−100.0 (3)	O3^i^—Cr1—N4—C9	98.2 (7)
N3—Pb1—O1—C4	−9.5 (3)	N1—Cr1—N4—C9	−146.2 (3)
O3—Pb1—O1—C4	−164.7 (3)	O1—Cr1—N4—C7	−169.9 (3)
O2^i^—Pb1—O1—Cr1	62.91 (14)	O2—Cr1—N4—C7	7.1 (3)
N3—Pb1—O1—Cr1	153.37 (16)	O3—Cr1—N4—C7	−85.6 (3)
O3—Pb1—O1—Cr1	−1.78 (10)	O3^i^—Cr1—N4—C7	−18.0 (8)
O1—Cr1—O3—Cr1^i^	−99.92 (12)	N1—Cr1—N4—C7	97.6 (3)
O2—Cr1—O3—Cr1^i^	86.21 (12)	C6—N3—C3—C4	−173.7 (4)
O3^i^—Cr1—O3—Cr1^i^	0.0	C5—N3—C3—C4	65.9 (5)
N1—Cr1—O3—Cr1^i^	−29.3 (8)	Pb1—N3—C3—C4	−55.6 (4)
N4—Cr1—O3—Cr1^i^	169.31 (13)	Cr1—O1—C4—C3	−177.4 (3)
O1—Cr1—O3—Pb1	−1.74 (10)	Pb1—O1—C4—C3	−16.7 (5)
O2—Cr1—O3—Pb1	−175.61 (11)	N3—C3—C4—O1	52.2 (5)
O3^i^—Cr1—O3—Pb1	98.18 (12)	C10—N4—C7—C8	90.1 (5)
N1—Cr1—O3—Pb1	68.9 (8)	C9—N4—C7—C8	−153.7 (4)
N4—Cr1—O3—Pb1	−92.51 (12)	Cr1—N4—C7—C8	−33.4 (5)
O2^i^—Pb1—O3—Cr1	−95.92 (12)	N4—C7—C8—O2	52.8 (6)
O1—Pb1—O3—Cr1	1.63 (10)	C7—C8—O2—Cr1	−45.9 (5)
N3—Pb1—O3—Cr1	−28.53 (17)	C7—C8—O2—Pb1^i^	165.2 (3)
O2^i^—Pb1—O3—Cr1^i^	6.24 (9)	O1—Cr1—O2—C8	48.5 (12)
O1—Pb1—O3—Cr1^i^	103.79 (11)	O3—Cr1—O2—C8	117.4 (3)
N3—Pb1—O3—Cr1^i^	73.63 (15)	O3^i^—Cr1—O2—C8	−163.7 (3)
O2^i^—Pb1—N3—C6	−124.8 (3)	N1—Cr1—O2—C8	−71.6 (3)
O1—Pb1—N3—C6	149.9 (3)	N4—Cr1—O2—C8	21.1 (3)
O3—Pb1—N3—C6	178.2 (3)	O1—Cr1—O2—Pb1^i^	−156.1 (10)
O2^i^—Pb1—N3—C5	−0.3 (3)	O3—Cr1—O2—Pb1^i^	−87.20 (13)
O1—Pb1—N3—C5	−85.5 (3)	O3^i^—Cr1—O2—Pb1^i^	−8.32 (13)
O3—Pb1—N3—C5	−57.3 (3)	N1—Cr1—O2—Pb1^i^	83.85 (15)
O2^i^—Pb1—N3—C3	118.4 (2)	N4—Cr1—O2—Pb1^i^	176.47 (16)

Symmetry code: (i) −*x*+1, −*y*+1, −*z*+1.

### Hydrogen-bond geometry (Å, º)

**Table:**

*D*—H···*A*	*D*—H	H···*A*	*D*···*A*	*D*—H···*A*
O3—H3···N2	0.82	1.95	2.757 (6)	169
